# Solvent/Detergent Virally Inactivated Serum Eye Drops Restore Healthy Ocular Epithelium in a Rabbit Model of Dry-Eye Syndrome

**DOI:** 10.1371/journal.pone.0153573

**Published:** 2016-04-21

**Authors:** Ching-Li Tseng, Zhi-Yu Chen, Ting-Yi Renn, Shun-Hung Hsiao, Thierry Burnouf

**Affiliations:** Graduate Institute of Biomedical Materials and Tissue Engineering, College of Biomedical Engineering, Taipei Medical University, Taipei, Taiwan; Boston University School of Medicine, UNITED STATES

## Abstract

Application of autologous serum eye drops (SEDs) is a recognized means to treat severe dry-eye syndrome (DES). Due to the inconvenience and difficulty of preparing SEDs from some patients, producing SEDs from allogeneic blood donations is gaining popularity. A major safety concern associated with allogeneic blood is virus transmission. We therefore herein evaluated the possibility of applying a solvent/detergent (S/D) treatment to inactivate viruses and studied the impacts of such treatment of SEDs to resolve DES in a rabbit model. Sera prepared from the blood of five rabbits were pooled and divided into two sub-pools. One was untreated (SEDs), while the other was virally-inactivated with 1% Tri-n-butyl phosphate/1% Triton X-45 at 31°C for 1 h (S/D-SEDs). DES was induced in rabbits using 0.1% benzalkonium chloride (BAC). Rabbits were divided into five groups of two rabbits each. One group was untreated (control), three were treated twice daily for 3 weeks using PBS, SEDs, or S/D-SEDs, and the last received an additional 0.1% BAC (as the negative control). The DES condition was determined by measuring aqueous tear secretion (Schirmer’s test), corneal fluorescein staining, a corneal histologic examination, TUNEL stain apoptosis, and corneal inflammatory marker (tumor necrosis factor-α, interleukin (IL)-1β, IL-8, and IL-6) expressions. We first confirmed that SEDs and S/D-SEDs had similar protein profiles and transforming growth factor (TGF)-β contents. Animal experiments showed that tear secretion did not significantly differ between the SED and S/D-SED groups but was significantly higher than in the PBS group. Eye fluorescein staining revealed dramatic improvements in epithelial defects in groups treated with SEDs or S/D-SEDs, and hematoxylin/eosin staining revealed microscopic epithelial layers similar to those of the untreated controls. Inflammatory markers and TUNEL studies showed that healthy epithelium had been restored in groups treated with SEDs or S/D-SEDs. In conclusion, this preclinical study supports the possibility of using S/D virally inactivated SEDs to treat DES and restore a normal epithelium.

## Introduction

Dry-eye syndrome (DES) is a multifactorial disease of the ocular surface characterized by rapid evaporation and insufficient production of tears. DES can lead to inflammation of the ocular surface, damage to the cornea, and conjunctiva, thereby impacting visual function and affecting the quality of life. DES has a prevalence of 5% to >35%, which increases with age [1,2]. Its severity is worsened by pathological conditions such as Sjögren's syndrome, which induces disruption of the ocular surface epithelium [3], or chronic graft-versus-host disease (GVHD) [4]. Regular use of artificial tears, anti-inflammatory drops, or punctal plugs can provide release, that is, however, only temporary and often induces ocular side effects.

Serum eye drops (SEDs) made from human blood have recently emerged as an alternative and apparently more-efficient method than conventional treatment to improve the ocular surface health, tear film stability, and subjective comfort in refractory DES [5–11]. The therapeutic benefits of SEDs are probably multifactorial, and can be explained by a composition that shares similarities with that of tears [5], including antimicrobial components, various platelet-derived growth factors, fibronectin, and vitamins [3,12]. Albumin, a major serum component, may also contribute to ameliorating ocular surface damage [13].

SEDs are currently most often prepared from autologous blood donations within hospitals or blood establishments [14,15]. However, preparing autologous serum requires particular attention and repeated blood samplings from patients, which is a constraint, and thus is potentially impossible in some clinical situations where patients are not in a condition or willing to donate blood. When an autologous source is unavailable, allogeneic serum, made from the serum of blood transfusion donors, is the next obvious option that in therapeutic terms appears equally efficient as autologous SEDs [16–18]. Allogeneic serum can be prepared by some blood establishments in a production setting that should comply with good manufacturing practice (GMP) principles [19,20], to ensure product standardization and quality [20]. The other substantial advantage of allogeneic SED is its off-the-shelf availability.

The main potential safety concern associated with allogeneic SEDs is, based on the historical experience with transfused blood products, the risk of infection by blood-borne pathogens, particularly viruses, and is a concern for clinicians and patients [17]. Although there is no data available indicating the extent of the possible infectious risks associated with the use of allogeneic topical SEDs, viral safety strategies of all therapeutic blood products should involve careful donor screening and blood donation testing for human immunodeficiency virus (HIV), and hepatitis B and C viruses [21]. However, information from the transfusion of intravenous blood components indicates that these measures do not prevent residual risks from infectious window-phase donations, when screening tests are not reactive but the donor is actually infectious. In addition, the blood supply is regularly exposed to emerging viruses, such as Dengue virus, Chikungunya virus, Ebola virus, Middle East respiratory syndrome coronavirus, and Zika virus that can be transmitted by blood products [22]. Currently, a higher margin of safety of blood components against a wide range of viruses can only be achieved by implementing dedicated virus pathogen inactivation treatments [23]. Treatments based on photochemical pathogen virus inactivation have recently been developed for plasma and platelet blood components [24], but not for serum. Their proprietary nature restricts their evaluation of new products like SEDs. In contrast, solvent-detergent (S/D) treatment that was developed in the late 1980s is in the public domain. S/D treatment is already applied to a wide range of biopharmaceutical preparations and plasma products, and is highly efficient against lipid-enveloped viruses [21]. We report here for the first time application of S/D treatment to the viral inactivation of SEDs. We also compared the safety and efficacy of S/D-treated and untreated rabbit SEDs in a DES rabbit model to determine whether S/D treatment is a realistic, safe option for viral inactivation of allogeneic SEDs.

## Materials and Methods

### Animal experiments and ethical statements

The experimental protocol was approved by the Institutional Animal Care and Use Committee of Taipei Medical University (IACUC approval no. LAC-100-0165). Male New Zealand rabbits purchased from Animal Health Research Institute, Council of Agriculture, Executive Yuan, Taiwan, weighing 2.5~3.5 kg with no signs of ocular inflammatory or gross abnormalities were used. The animals were housed in standard cages in a light-controlled room and were given food and water ad libitum. All examinations or surgical procedures on the rabbits were performed under general anesthesia administered via an intramuscular injection of a 1:2 mixture of Zoletil 50 (Virbac Anial Health, Nice, France) and 2% Rompun solution (Bayer, Kyonggi-do, Korea).

### Study design

**Fig 1:**
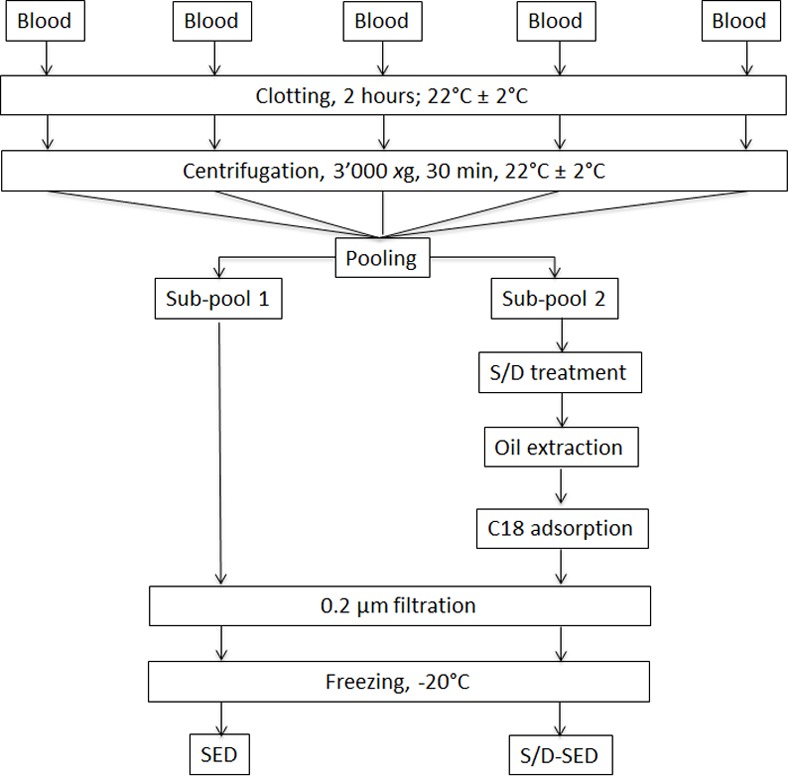
Preparation method of serum eye drops (SEDs) and solvent/detergent-treated (S/D)-SEDs. For experimental details see the "Materials and Methods" section.

The study design is summarized in Fig 1 and explained below.

### Preparation of blood fractions

#### Rabbit blood collection and preparation of serum

Blood was collected from five rabbits by cardiac puncture. Blood from each rabbit (approximately 80~100 mL) was allowed to clot for 2 h to generate serum and was then centrifuged at 3000 x*g* for 30 min at 22 ± 2°C. The supernatant serum was recovered, pooled (220 mL in total), and divided into two fractions. An aliquot of 90 mL was filtered on a 0.2-μm filter to remove bacteria and insoluble materials. Serum was dispensed into Eppendorf tubes (0.5 mL/tube) and frozen at -20°C. The remaining 130 mL of serum was subjected to S/D treatment in a polyvinyl chloride bag using 1% (v/v) tri-n-butyl phosphate (TnBP; Merck, Darmstadt, Germany) and 1% (v/v) Triton X-45 (Sigma, St. Louis, MO, USA) at 25°C for 1 h under constant gentle stirring. The mixture was extracted and clarified by adding 10% (v/v) soybean oil (Sigma), with vigorous shaking for 30 s, mild shaking for 15 min, and decantation for 40 min at 22 ± 2°C as in our previous research [25]. The clarified bottom protein layer was recovered, and the upper oil layer was discarded. Residual S/D agents were further removed by treating the protein phase with hydrophobic interaction chromatographic adsorption at a ratio of 6 mL per 0.5 g of sorbent (octadecyl C18; 125 Å; porosity; 55~105 μm; Waters, Guyancourt, France) for 1 h at 22 ± 2°C. The mixture was centrifuged at 6000 x*g*, and the supernatant (S/D-serum) was recovered, sterile-filtered on a 0.2-μm filter, and frozen at < -20°C [26].

#### Serum characterization

Total proteins were quantified by the Bradford assay. Sodium dodecylsulfate polyacrylamide gel electrophoresis (SDS-PAGE) under non-reducing and reducing conditions was performed using 4%~12% gradient gels, reagents, electrophoretic systems from Invitrogen (Carlsbad, CA, USA), and a prestained protein molecular weight standard (Novex Sharp, Invitrogen) [27]. The transforming growth factor (TGF)-β content was determined with an enzyme-linked immunosorbent assay (ELISA; Rabbit TGF-β1; Shanghai Yehua Biological Technology, Shanghai, China), and epidermal growth factor (EGF) with a rabbit EGF sandwich ELISA kit (my.biosource.com, San Diego, CA, USA).

### DES induction

Rabbits were housed at 23 ± 2°C, a relative humidity of 60% ± 10%, and an alternating 12-h light-dark cycle (06:00~18:00). DES was induced as described in previous studies [28,29] with slight modifications [30]. Briefly, eyes were topically treated with 20 μl of eye drops containing 0.1% benzalkonium chloride (BAC; Sigma-Aldrich, St. Louis, MO, USA) by triple-daily dripping (10:00, 14:00, and 18:00) for 4 weeks. Clinical observations, Schirmer’s test, and fluorescein staining were performed, and the central corneal thickness (CCT) was determined before and after DES was induced by BAC treatment in rabbits.

### Topical delivery of platelet lysates for DES treatment

Following BAC treatment, 10 rabbits were randomly divided into five groups. Four groups were treated twice daily (10:00 and 18:00) for 3 weeks using various formulations: (1) phosphate-buffered saline (PBS); (2) serum; (3) S/D-serum; and (4) 0.1% BAC for an additional 3 weeks (negative control). A fifth group with no treatment served as a control. After 3 weeks of treatment, the DES condition of the rabbits was examined as described below.

### Examination for DES conditions

#### Measurement of aqueous tear production

Aqueous tear secretion was measured by Schirmer’s strips (Madhu Instruments, New Delhi, India) [28,31]. Briefly, rabbits underwent general anesthesia for immobilization (intramuscular injection of 1~1.5 ml of a 1:2 mixture of Zoletil 50 and 2% Rompun solution). The same operator conducted the tests at defined time points under a standard controlled environment. After topical administration of 0.5% Alcaine® (Alcon, Puurs, Belgium), the lower eyelid were slightly pulled down, and the paper strip for Schirmer’s test was placed on the palpebral conjunctival vesica, located near the junction of the middle and outer third of the lower lid. After 5 min, the length of the wetting on the paper strip was read, and data were expressed in millimeters. Each eye was tested twice at 30-min intervals, and the average length of paper wetting was calculated.

#### Fluorescein (FL) staining

Corneal FL staining was performed before and after DES induction. The therapeutic effect was also evaluated after 3 weeks of treatment. FL paper strips (Haag-Streit AG, Koniz/Bern, Switzerland) were smeared directly on the cornea by letting the dye drop onto the conjunctival sac [28,31]. The ocular surface was examined and graded under a slit-lamp microscope with a cobalt-blue filter (Topcon Medical Systems, Oakland, NJ, USA).

#### Histological staining of the cornea

Animals were euthanized with an overdose of anesthetics (ketamine: zylazine of 120: 15 mg/kg), and the corneas were collected. One cornea was divided into two equal parts; one part was fixed in a 3.7% formaldehyde buffer solution for at least 24 h for histological observations, and the other one without fixation was frozen at -80°C for protein extraction, as described in the next section. Fixed specimens were embedded in paraffin. Sections of corneas were stained with hematoxylin and eosin (H&E; Sigma) for histologic examination. Terminal deoxynucleotidyl transferase deoxyuridine triphosphate nick end labeling (TUNEL) staining was performed to examine apoptosis of the corneal epithelium [32,33] using an Apo-BrdU-IHC^TM^ In Situ DNA Fragmentation Assay Kit following the supplier’s recommendations (BioVision, Milpitas, CA, USA).

#### Quantification of inflammatory cytokines in the cornea

Thawed corneas were weighed and separately chopped into small pieces. Liquid nitrogen was added to immerse the tissue for solidification, and it was then ground into a powder. This step was repeated three times. The tissue extraction solution containing 99% T-PER Tissue Protein Extraction Reagent and 1% Halt^TM^ Protease and Phosphatase Inhibitor Cocktail (both from Thermo Fisher Scientific, Rockford, IL USA) were added to the tissue at a ratio of 15 mL per 1 g of cornea. The tissue-extraction mixture was kept at -20°C for 10 min, and milled again at room temperature for another 5 min. This protocol was repeated five times. The tissue-extraction mixture was centrifuged at 10^4^ x*g* for 10 min at 4°C to remove tissue debris. The tissue extract was collected and analyzed by a Coomassie protein assay (Thermo Fisher Scientific) to normalize the protein content of the samples for subsequent cytokine analysis by an ELISA. Total protein (15 μg in 100 μL) was loaded into 96-well plates for the ELISA. Rabbit tumor necrosis factor (TNF)-α was determined using a DY5670 kit from R&D Systems (Minneapolis, MN, USA), and rabbit interleukin (IL)-1β, IL-8, and IL-6 were respectively determined using MBS725024, MBS2507990, and MBS812867 kits from MyBioSource (San Diego, CA, USA) following the suppliers’ instructions.

### Statistical analysis

Values are presented as the mean ± standard deviation (SD) or standard error of the mean (SEM). Statistical differences between groups were assessed using one-way analysis of variance (ANOVA) post hoc tests and by Tukey’s test using SPSS 17.0 (SPSS, Inc., Chicago, IL, USA). A probability (*p*) value of less than 0.05 was considered statistically significant.

## Results

### Characterization of SEDs and S/D-SEDs

**Fig 2:**
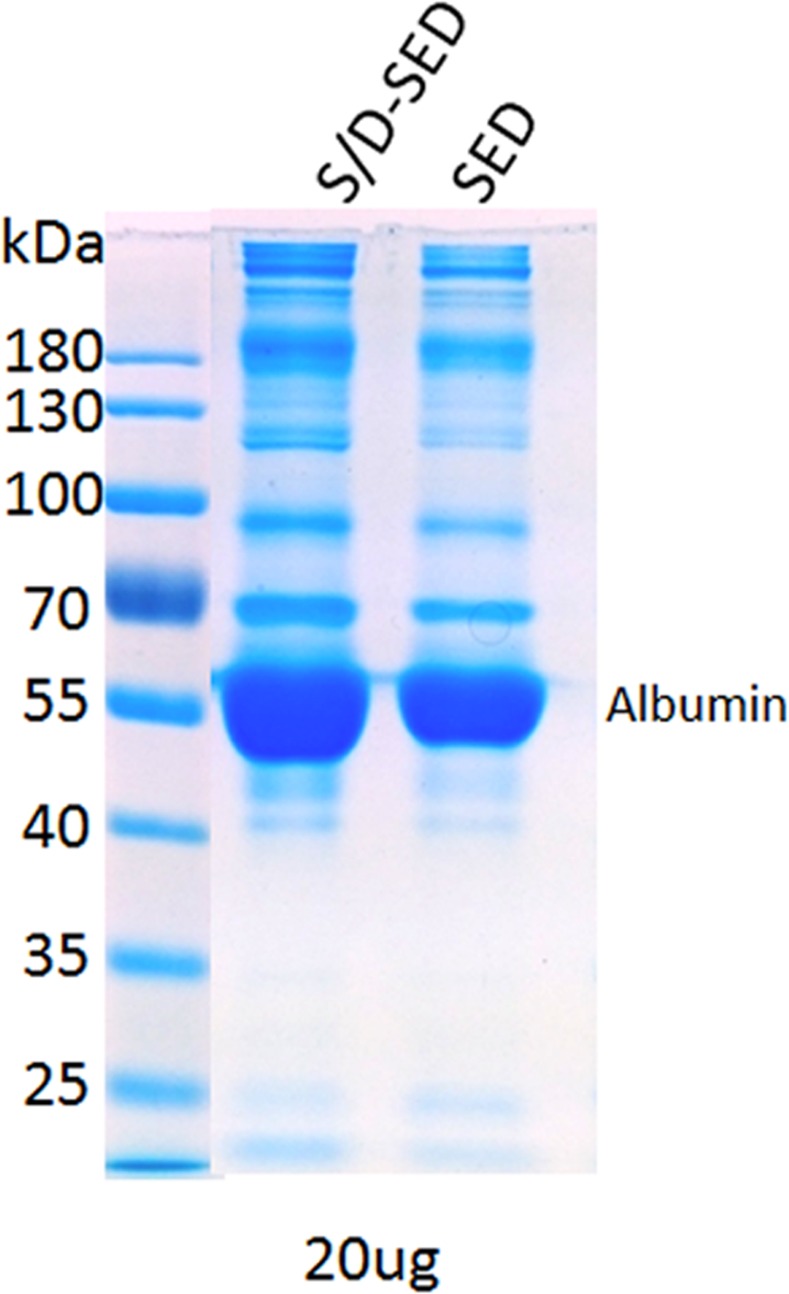
SDS-PAGE under non-reducing conditions. Molecular weight protein markers (left lane). Total protein content in each lane was normalized to 20 μg. Abbreviations: kDa: kilodaltons; SEDs: serum eye drops; S/D-SEDs: solvent/detergent-treated serum eye drops.

Protein contents of SEDs and S/D-SEDs were 83 and 68 mg/ml, respectively. SDS-PAGE revealed no substantial differences in protein compositions regardless of the molecular mass, with an expected prominent band corresponding to albumin (Fig 2). TGF-β levels were 117 and 98 ng/ml in SEDs and S/D-SEDs, respectively, whereas EGF was undetectable (< 32 pg/ml) in both preparations.

### DES rabbit model data

#### DES induction and treatment

The timeline from DES induction to treatment was as follows. First, rabbits were treated with 0.1% BAC three times daily for 4 weeks to establish DES. Next, rabbits with established DES were treated with SEDs or S/D-SEDs twice daily for 3 weeks.

#### Schirmer’s test

**Fig 3:**
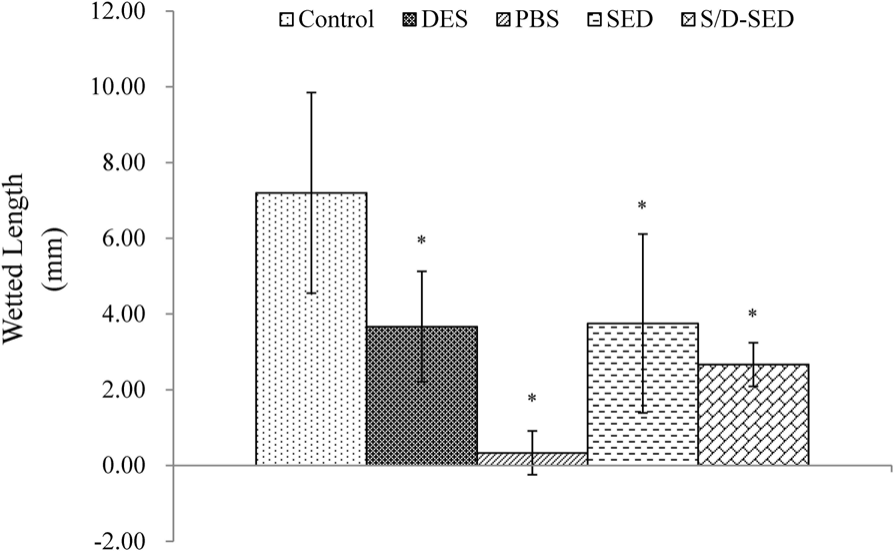
Schirmer’s test. Data from all groups after 3 weeks of treatment. Data are shown as the mean ± SD. * *p<*0.05 significantly from the control group (by One way ANOVA-Tukey’s multiple comparison. Duplicate experiments. Abbreviations: SEDs: serum eye drops; S/D-SEDs: solvent/detergent-treated serum eye drops.

Tear production was examined after the 3-week eye drop treatment period. Tear secretion rapidly decreased in eyes treated with 0.1% BAC (which mimics DES) and PBS, compared to normal eyes. All groups were significantly difference from control group (normal eye, *p<0.05). Compared to the DES group, tear production did not significantly differ (*p* < 0.05) in the group treated with SEDs or S/D-SEDs, but was still lower than that of the normal group (Fig 3).

#### FL staining

**Fig 4:**
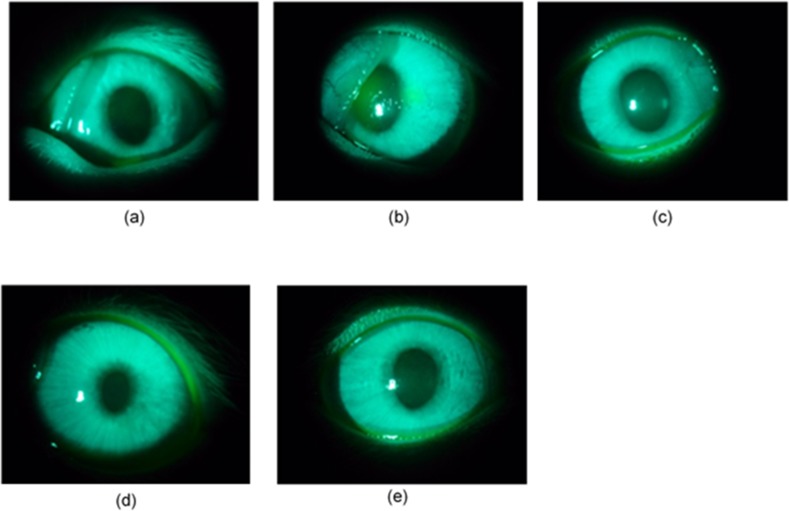
Slit-lamp photographs of rabbit eyes in each group after fluorescein staining. The epithelial defect area appears as green patches. (a) Control: a non-treated eye with no fluorescent pigment, (b) dry-eye syndrome (DES; 0.1% BAC); (c) PBS-treated eyes; (d) serum eye drops (SEDs) and (e) solvent/detergent-treated (S/D)-SEDs. A dramatic improvement of the epithelial defect was observed in the two groups treated with SEDs and S/D-SEDs, in which treated eyes were similar to the control groups and with no staining.

After the 3-week treatment period, the ocular surface of all experimental eyes was stained with FL and examined under a slit-lamp microscope. No obvious staining was observed in eyes of normal animals (Fig 4A). In contrast, dark-green staining was observed in the center of corneas of eyes treated with 0.1% BAC (Fig 4B). Opaque zones in the center of corneas were observed in eyes treated with PBS (Fig 4C). No staining was observed in corneas of eyes treated with either SEDs or S/D-SEDs (Fig 4D and 4E).

#### Histological examination

**Fig 5:**
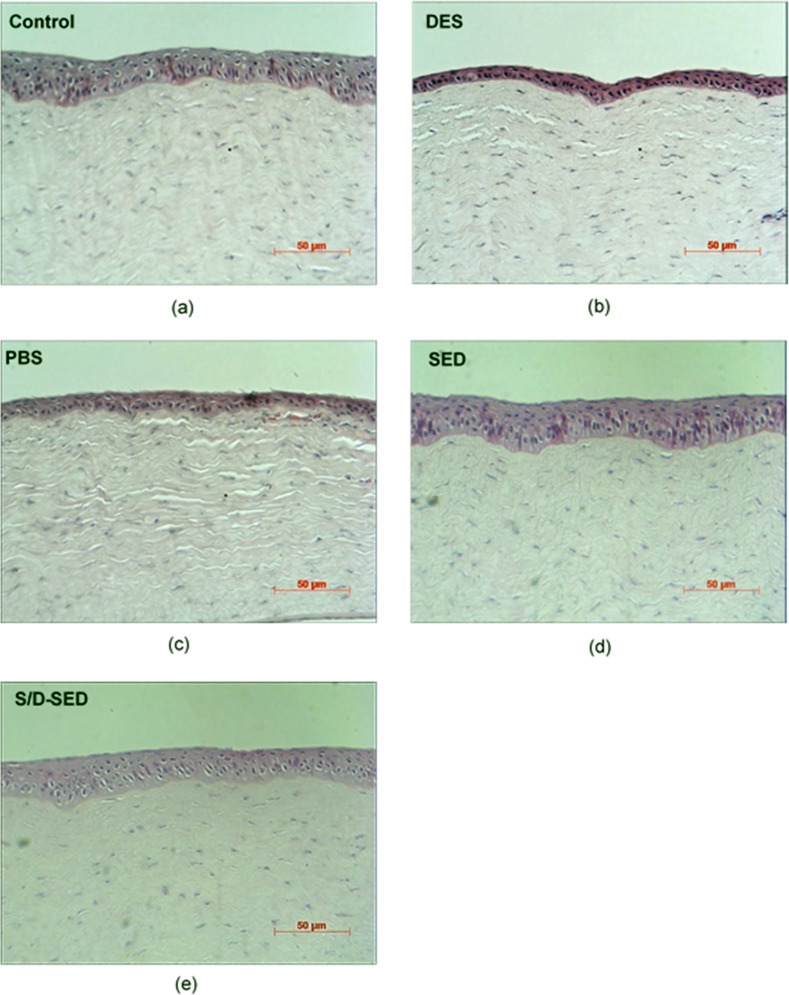
Representative pictures of HE staining of corneas from each of the five groups. (a) Normal corneas (control): three to five epithelial layers can be observed. Damage to the epithelium was observed in (b) dry-eye syndrome (DES; 0.1% BAC) and (c) phosphate-buffered saline (PBS)-treated group: the layered structured was destroyed and sponge-like stroma were found; (d) serum eye drops (SEDs) and (e) solvent/detergent-treated (S/D)-SEDs were similar to control groups with multilayered epithelium in the apical part.

Examination under a light microscope showed that normal corneas (control) had three to five layers of epithelial cells and dense collagen fibrils in the stroma (Fig 5A). Corneal sections in the 0.1% BAC-treated group showed a thinner corneal epithelium with only two or three layers and loose collagen fibrils in the stroma (Fig 5B). Corneal sections of the PBS group had thinner corneal epithelia and loose stroma (Fig 5C). In contrast, rabbits treated with SEDs and S/D-SEDs had normal corneal epithelia with a normal number of layers and thicknesses (Fig 5D and 5E).

**Fig 6:**
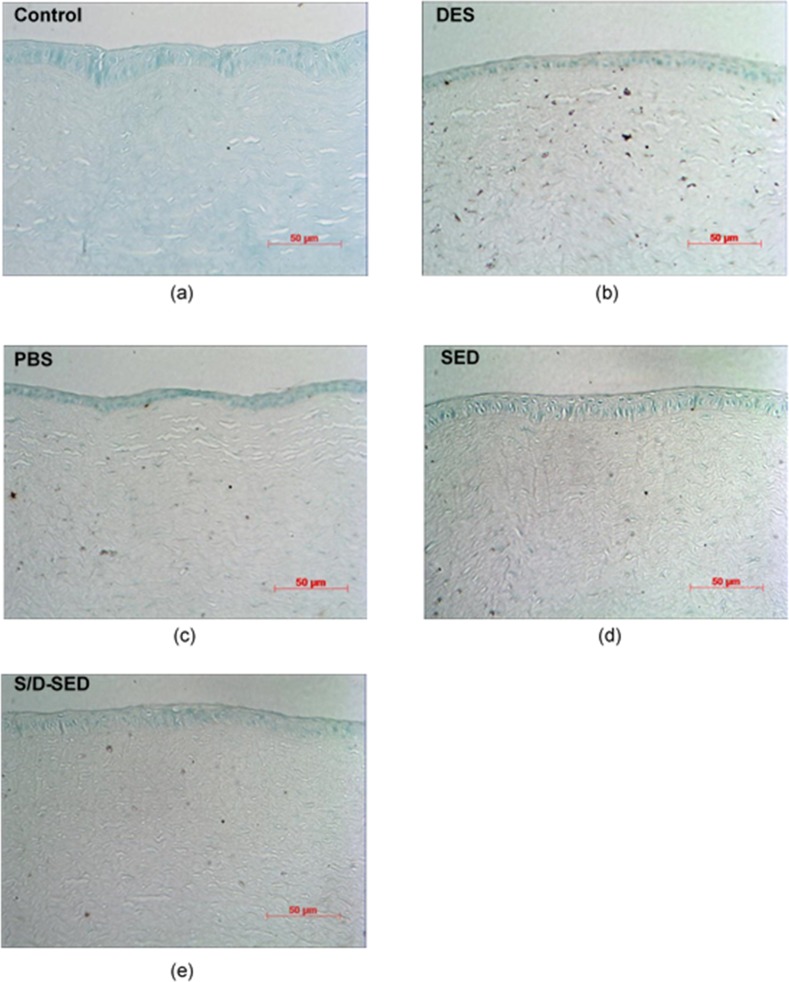
TUNEL staining of corneal sections from each group. (a) Control, (b) dry-eye syndrome (DES; 0.1% BAC), and (c) phosphate-buffered saline (PBS)-treated groups showed positive staining (blue) representing serious damage to the epithelium layer; (d) serum eye drop (SED) and (e) solvent/detergent-treated (SD)-SED groups were similar to the control groups with a healthy epithelium in the apical part.

TUNEL is a common method for detecting DNA fragmentation that results from apoptotic signaling cascades. In this assay, cells that had suffered severe DNA damage stained dark brown. Normal eyes (control) showed nearly no TUNEL-positive cells (Fig 6A). In contrast, eyes treated with 0.1% BAC (DES) showed large numbers of apoptotic cells in the corneal basal epithelium and stroma (Fig 6B), whereas those treated with PBS also showed apoptotic cells in the corneal epithelium and stroma (Fig 6C). Eyes treated with SEDs or S/D-SEDs showed fewer TUNEL-positive cells (Fig 6D and 6E).

#### Inflammatory markers

**Fig 7:**
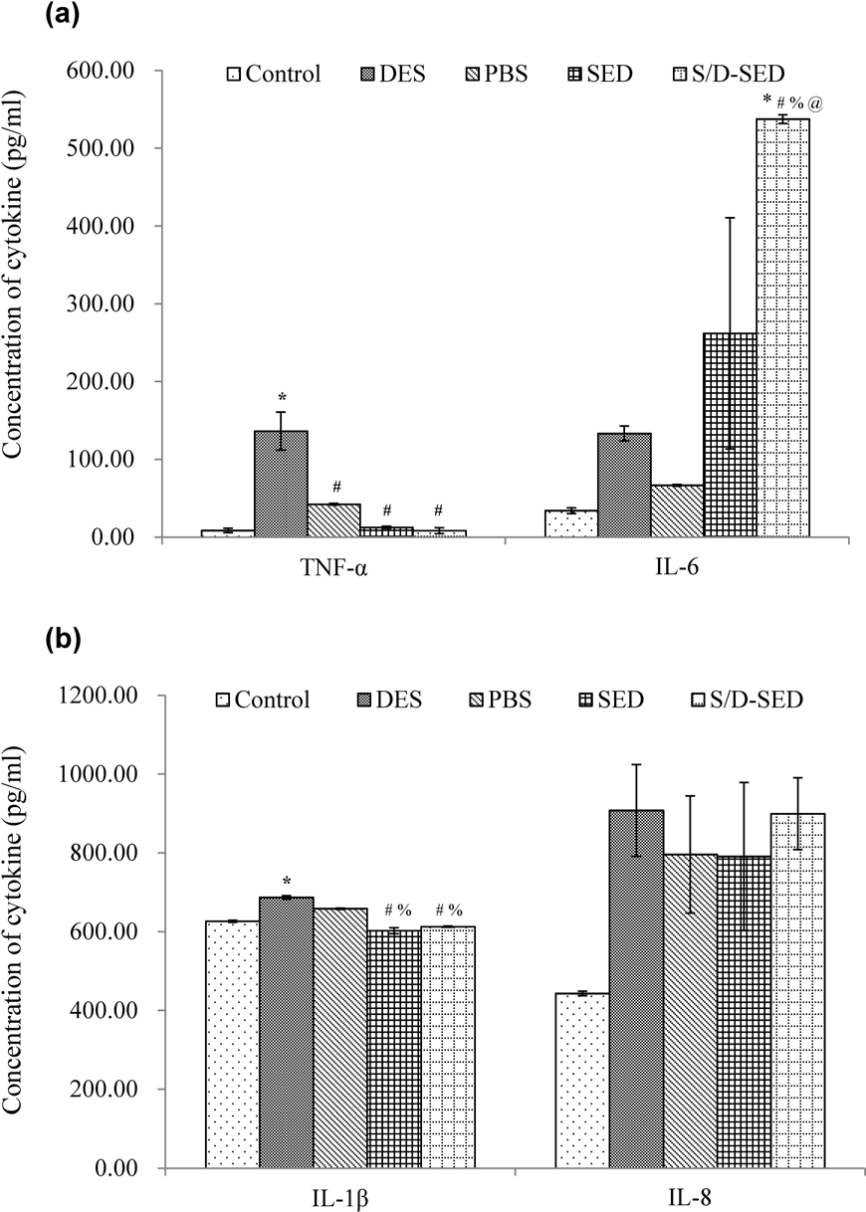
Variation in tumor necrosis factor (TNF)-α, interleukin (IL)-6, IL-1β, and IL-8 inflammatory marker expressions in corneas exposed to different treatments. Data are expressed as the mean ± SD (error bars). * *p*<0.05 versus control group; # *p*<0.05 versus DES; % *p*<0.05 versus PBS; @ *p*<0.05 versus SED (One way ANOVA—Tukey’s multiple comparisons).

Inflammation of the corneas was assessed by ELISA measurements of the expressions of inflammatory cytokines in response to various treatments (Fig 7). Higher levels of TNF-α, IL-6, IL-1β, and IL-8 were detected in the 0.1% BAC (DES)-treated group, as expected, since this model mimics inflammatory conditions associated with DES (^*^p<0.05). Concentrations of TNF-α and IL-1β were significantly reduced in the SED- and S/D-SED-treated groups compared to the DES group (^#^*p*<0.05). The concentration of IL-8 in inflamed corneas treated with SEDs or S/D-SEDs was higher than that in the normal control group but lower than that of the DES-treated one, evidencing only partial relief of the inflammatory conditions. The IL-6 concentration in all groups was much higher than that in normal corneas. Therefore, according to these results, using SEDs or S/D-SEDs for topical delivery to treat DES rabbits could selectively inhibit inflammatory cytokines in the eyes.

## Discussion

DES is a frequent disorder prevalent in the elderly and in patients with Sjögren's syndrome [3] or chronic GVHD [4]. DES symptoms include pain, irritation, itching, burning, and grittiness which all have serious adverse impacts on daily life activities [34,35]. In addition, DES is associated with inflammation of the ocular surface [31,36,37].

Artificial tears, anti-inflammatory drops, and punctal plugs are conventional treatments, but they provide only partial benefits in addition of having side effects. Eye drops made of human serum have emerged as an alternative and probably superior treatment of DES [6,11,38,39]. SED preparations from autologous sources have so far been used most frequently [5,6,14,15], and standardized preparation procedures using a closed cascade filtration system were developed [40]. However, there is a trend towards using allogeneic, off-the-shelf SEDs prepared under controlled GMP conditions by blood establishments [20,41]. Still, a legitimate concern associated with the use of allogeneic SEDs is the risk of viral contamination. Although donor screening and donation testing can provide a high viral safety margin, risks are not completely eliminated, even in developed economies with access to the most efficient nucleic acid viral testing procedures, due to residual window-phase donations of HIV, hepatitis B virus, and hepatitis C virus [42,43]. In addition, world populations, due to increasing travel and migration and global warming, are regularly exposed to new emerging viruses [44]. Such viruses, when collected from asymptomatic blood donors and untested, could potentially be transmitted to SED recipients, although the extent of the risks of viral infections is still currently unknown. It has been shown, for instance, that respiratory syncytial virus can use the eye as an entry pathway, replicate robustly, and eventually migrate to the lung [45]. Thus, optimal viral safety of allogeneic SEDs requires implementation of additional safety measures such as dedicated pathogen inactivation treatment implemented by manufacturers. Among pathogen-inactivation procedures theoretically applicable to SEDs, S/D was selected here for SED as it is very efficient against lipid-enveloped viruses [46], which represent major threats to the safety of blood-derived products. We therefore evaluated S/D treatment as a possible tool to enhance the safety of SEDs. We elected to treat SEDs with a combination of 1% TnBP and 1% Triton X-45 at 31°C. These conditions applied to plasma and cryoprecipitates are known to inactivate, within a few minutes, over 5 log of HIV, porcine pseudorabies, bovine viral diarrhea virus [47], hepatitis C virus [48], and Dengue virus [49]. S/D treatment affects viruses by disrupting their lipid envelope, not by removal. It can also inactivate some bacteria [50] as well as parasites and white blood cells. Such S/D treatment does not alter the plasma protein functionality or content, including albumin [47], which was shown to contribute to apoptosis suppression of ocular epithelium cells in a dry-eye model [51]. In addition, and quite importantly, this S/D treatment was shown not to alter the antimicrobial [50] or anti-inflammatory [52] activities of plasma components. We showed that the procedure based on soybean oil extraction and C18 hydrophobic interaction chromatography efficiently removes S/D agents [53,54] thereby avoiding toxicity. Therefore, this treatment is capable of providing a high safety margin against lipid-enveloped viruses without impairing key functional features of SEDs. There is, however, some quantitative loss of proteins associated with the S/D treatment steps, in particular the oil extraction and the C18 adsorption.

To compare the efficacy of SEDs, whether S/D treated or not, to relieve DES we used a preclinical rabbit model where DES was induced by the topical administration of BAC, a preservative of ophthalmic drugs, that worsens preexisting dry-eye conditions [55,56]. BAC alters both the cornea and conjunctiva [57], leading to physiological changes mimicking DES symptoms in humans [58]. The large ocular surface of the rabbit eye permits adequate monitoring of tear production and more-efficient histological and biochemical analyses compared to mice [59]. Our animal model was inspired by previously published methods [29] but was modified by exposing the eyes to 0.1% BAC three times daily for 4 weeks followed by 3-week treatment with 0.1% BAC or SEDs, resulting in a stable DES state that did not spontaneously recover after discontinuing BAC [30]. Our results demonstrated that pathogen inactivation did not impair the efficacy of SEDs to relieve DES symptoms in this rabbit model based on the following relevant evaluation criteria: restoration of tear production, FL staining observations, histological examinations, and quantification of inflammatory markers.

In conclusion, our data support the possibility of performing pathogen inactivation treatment of SEDs using S/D treatment. These data, which should be confirmed using human SEDs, open perspectives for the development of standardized pooled allogeneic SEDs.

## Abbreviations

BAC: benzalkonium chloride
CCT: central corneal thickness
DES: dry-eye syndrome
EGF: epidermal growth factor
GMP: good manufacturing practices
GVHD: graft-versus-host-disease
IL: interleukin
SDS-PAGE: sodium dodecyl sulfate–polyacrylamide gel electrophoresis
SEDs: serum eye drops
S/D: solvent/detergent
TGF: transforming growth factor
TNF: tumor necrosis factor
TUNEL: terminal deoxynucleotidyl transferase deoxyuridine triphosphate nick end labeling
